# Early Alarm on the First Occurrence of the Southern Giant Hornet *Vespa soror* du Buysson, 1905 (Vespidae) in Europe

**DOI:** 10.1002/ece3.70502

**Published:** 2024-11-09

**Authors:** Omar Sánchez, Leopoldo Castro, Álvaro Fueyo, Yaisel J. Borrell, Andrés Arias

**Affiliations:** ^1^ Department of Organisms and Systems Biology (Zoology) University of Oviedo Oviedo Spain; ^2^ Environment and Sustainability Area TAXUS Medio Ambiente Oviedo Spain; ^3^ Av. Sagunto, 44 (6º‐5ª) Teruel 44002 Spain; ^4^ Department of Functional Biology (Genetics) University of Oviedo Oviedo Spain

**Keywords:** exotic species, Iberian Peninsula, northern Spain, Vespinae

## Abstract

An eco‐monitoring program to assess the biodiversity of insects affected by yellow‐legged hornet (*Vespa velutina*) trapping in the north of the Iberian Peninsula (Spain) revealed the first occurrence of the southern giant hornet *Vespa soror* (Hymenoptera, Vespidae) on the European continent. We present a detailed characterization, combining morphological characteristics and molecular tools for genetic identification, as well as key information on its identification with respect to other hornets found on the Iberian Peninsula. We discuss the most plausible pathways and vectors of introduction, its potential invasiveness, and subsequent impacts on host localities. Our preliminary results raise concerns about the potential threat of *V. soror* to human health and ecosystem dynamics, as it is a highly predatory species on other insects and even small vertebrates. Finally, this study confirms once again the usefulness of studying insects trapped in such traps for rapid response and early detection of inland invasive species. We also propose a common Spanish name for the species, “avispón sóror”.

## Graphical abstract

First occurrence of the Asian southern giant hornet Vespa soror in Europe. We present a detailed description, combining morphological and molecular traits, as well as key information about its identification and current distribution. We discuss the most plausible pathways and vectors of introduction, its potential invasiveness, and subsequent impacts on host localities.

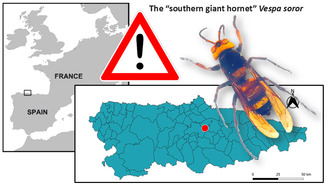

## 1 Introduction

The genus *Vespa* Linnaeus, 1758, belonging to the family Vespidae and the subfamily Vespinae, includes eusocial hymenopterans commonly known as hornets (Noll et al. 2020). In the last decades, globalization has made it easier for social Vespidae in general and the Vespinae in particular to introduce and spread accidentally in new areas and countries (Arca et al. 2015; Castro 2019; Lioy, Bergamino, and Porporato 2022; Otis, Taylor, and Mattila 2023). This introduction is favored mainly because the fertilized queens spend the meteorologically unfavorable part of the year hidden, using as shelter some material or container that humans can transport unnoticed far away from the insects' original locality (Arca et al. 2015; Castro 2019; Lioy, Bergamino, and Porporato 2022; Otis, Taylor, and Mattila 2023). Another key point for the successful introduction of vespines is the low number of individuals needed to establish themselves in new areas. Once the suitable environmental conditions are provided, it is only necessary for one or a few fertilized queens (depending on the species) to arrive safely and survive the most difficult period, until the hatching of the first workers, as happened in Europe with *Vespa velutina* Lepeletier, 1836 (Villemant et al. 2011; Monceau, Bonnard, and Thiéry 2014; Arca et al. 2015; Otis, Taylor, and Mattila 2023).

All species of the genus *Vespa* are endemic to Asia except for *Vespa crabro* Linnaeus, 1758, and *Vespa orientalis* Linnaeus, 1771, which are widespread in Europe and/or North Africa (Archer 2012; Smith‐Pardo, Carpenter, and Kimsey 2020). The Ibero‐Balearic fauna of the genus originally consisted of a single species, *Vespa crabro*, the “European hornet,” distributed over a large part of the Iberian Peninsula (Vega et al. 2022). However, in the past decade other species have arrived, among them *Vespa velutina* Lepeletier, 1836 (“Asian hornet”) (Castro and Pagola‐Carte 2010; López, González, and Goldarazena 2011; Grosso‐Silva and Maia 2012), *Vespa orientalis* (“oriental hornet”) (Hernández et al. 2013; Sánchez, Fajardo, and Castro 2019; Fajardo and Sánchez 2020), and *Vespa bicolor* Fabricius, 1787 (“black shield hornet”) (Castro 2019).

The “southern giant hornet”, *Vespa soror* Buysson, 1905, inhabits the warmer regions of Asia, including north‐eastern India (Arunachal Pradesh, Meghalaya, Nagaland), northern Myanmar, northern Thailand, Laos, northern Vietnam, and southern China (Yunnan, Fujian, Jiangxi, Zhejiang, Hunan, Guangdong, Hong Kong, and Hainan Island) (Lee 2009; Daglio 2019; Smith‐Pardo, Carpenter, and Kimsey 2020). It is one of the largest hornets, similar in size to its congener, the northern giant hornet (*Vespa mandarinia* Smith, 1852) (Kurzenko 1995; Girish‐Kumar and Srinivasan 2010; Lee 2010). *Vespa soror* is not believed to have any established introduced populations even in those regions where its congener *V. mandarinia* has been introduced. However, one queen was found at Vancouver Harbor, British Columbia, Canada, in 2019 (Kozak and Otis 2020; Bass, Needham, and Bennett 2022). In 2021, *V. mandarinia* was detected in British Columbia and across the Canada‐United States border in the Washington State (Bass, Needham, and Bennett 2022; Taylor et al. 2024), but this species seems to have vanished due to no detections in 2023 and 2024, and the provisional conclusion is that the introduction has failed through “some combination of human eradication efforts, Allee effects, ecological factors and chance” (Taylor et al. 2024).

In this paper we present the first European record of the southern giant hornet, *V. soror,* in Europe (from northern Spain), providing a genetic and morphological characterization of the specimens found. We present a detailed diagnosis and illustrations of the species in order to facilitate its differentiation from other native and introduced vespines of the Iberian Peninsula. A simplified pictographic key of all Iberian *Vespa* spp. is also provided. Finally, we discuss the most plausible introduction pathways and vectors, its current status, and the potential impacts it may generate if it becomes invasive in the receiving environment, and propose a new vernacular name for the species, “avispón sóror,” given some apparent disadvantages of the available Spanish names.

## 2 Materials and Methods

### 2.1 Specimen Collection, Morphological Analysis, and Taxonomic Procedures

**FIGURE 1:**
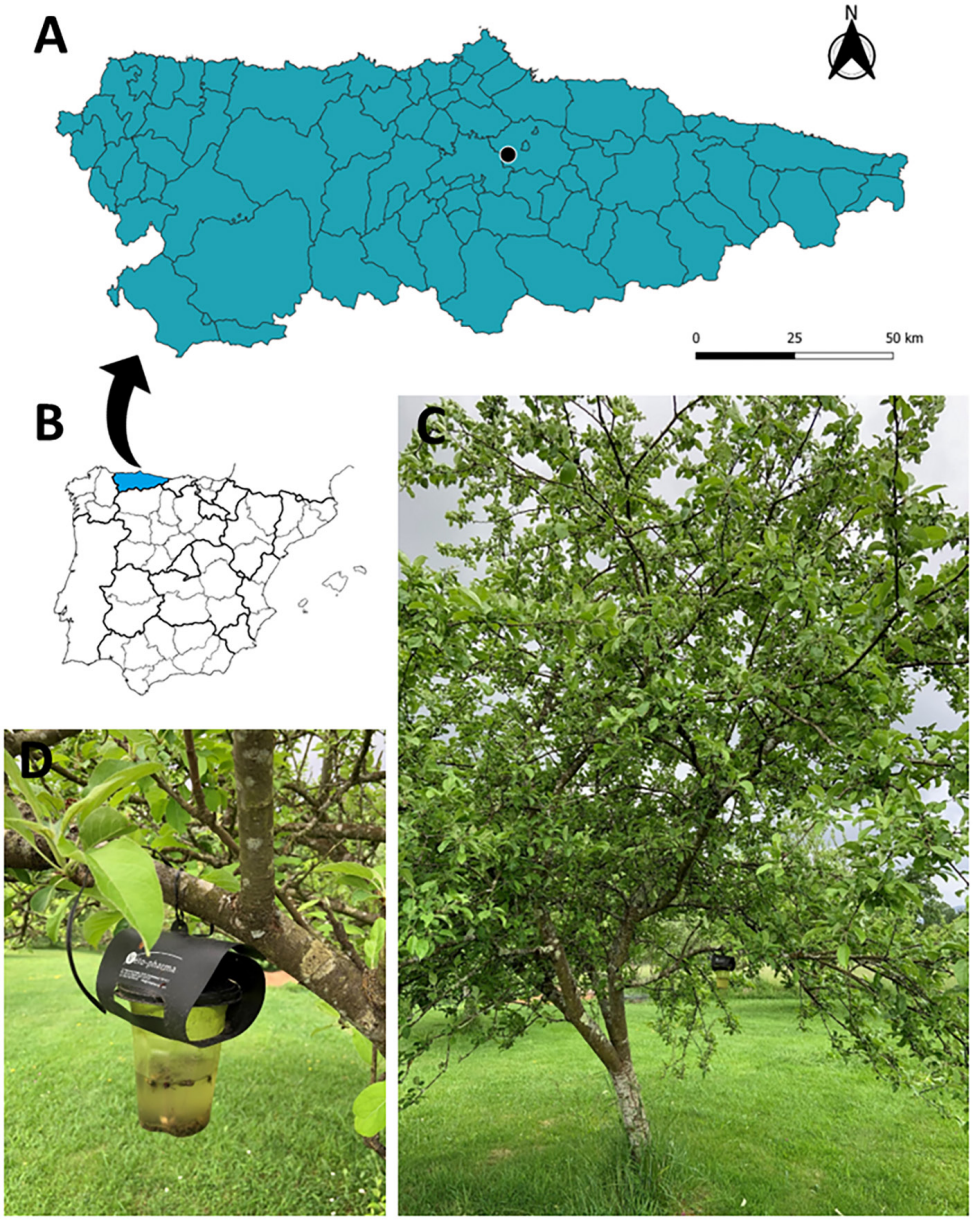
Geographical location where *Vespa soror* were found in the Principality of Asturias (a) and its situation in Spain (b). General view of the plot (c) and a detail of a “Vespa Catch” type trap (d) placed on the branch of an apple tree in the study location.

A total of four specimens of *V. soror* were collected in March 2022 and October 2023 at El Campo, Granda, Siero (43°22′36″ N, 5°45′41″ W) in the Principality of Asturias (northern Spain). The specimens were trapped in a *V. velutina* VespaCatch commercial trap made by Véto‐pharma (Palaiseau, France) filled with VespaCatch attractant (for additional Véto‐pharma information, see https://www.blog‐veto‐pharma.com/) (Figure 1). The specimens were prepared for preservation in situ, fixed in 70% ethanol for the genetic analysis, and lastly pinned. Specimens were examined under a dissecting stereomicroscope, Optika SZM‐2, 0.7–4.5×. Photographs were taken with a Canon EOS 1200D Digital SLR Camera with EF‐S 18–55 mm *f*/3.5–5.6 III Lens. The specimens were deposited in the collection of the Department of Organisms and System (BOS) of the University of Oviedo (https://bos.uniovi.es/).

### 2.2 DNA Extraction, PCR Amplification and Sequencing

DNA was extracted from 20 to 50 mg of ethanol‐preserved tissues from leg fragments from one specimen, using an E.Z.N.A. Tissue DNA Kit (Omega Bio‐tek) and following the manufacturer's protocol, and DNA samples were stored at −20°C. The mitochondrial cytochrome c oxidase subunit I (COI‐5P) gene was amplified by means of a polymerase chain reaction (PCR) in a total volume of 40 μL, using the universal primers LCO1490 and HCO2198 (Folmer et al. 1994). The reaction mixture contained 2.5 μL template DNA, 2.5 μM MgCl2, 0.25 mM each DNTP, 0.25 μM of each primer, 0.5 U Taq polymerase (GoTaq G2 Flexi DNA Polymerase), and the appropriate buffer at 1× final concentration. The PCR conditions used were an initial denaturation step of 95°C for 4 min, then 35 cycles of 95°C for 45 s, 48°C for 45 s, 72°C for 30 s, and finally an extension of 72°C for 7 min. The PCR product was checked via horizontal electrophoresis (2% agarose gel). Finally, the samples were sent for forward and reverse sequencing at MACROGEN (Madrid, Spain), using the standard Sanger sequencing method (Sanger and Coulson 1975).

### 2.3 Genetic Analysis

The forward and reverse sequences obtained by Sanger sequencing were edited using GENEIOUS PRIMER 2022.2.2 (https://www.geneious.com) for quality trimming and primer removal. Then they were aligned and manually checked to correct any possible incorrect base calling. After alignment and corrections, a consensus sequence was generated with the default parameters.

A preliminary genetic species identification was attempted using nBlast implemented in GENEIOUS PRIMER using the default values to search in GenBank databases. After this, a search in the identification engine from the Public Record Barcode Database of BOLD was also carried out (https://www.boldsystems.org/index.php/IDS_OpenIdEngine).

A phylogenetic analysis was conducted using 31 COI sequences from Genbank (Accessions in Supporting Information, SX). In addition to the new sequence obtained from the Spanish specimen, all sequences longer than 500 bp of *Vespa soror*, plus at least one sequence from each *Vespa* species with sequences available in the database and 4 outgroups, were used. The sequences were analyzed using RaxML software implemented in GENEIOUS PRIMER 2022.2.2. The ModelTest software included in the PAUP pipeline implemented in GENEIOUS PRIMER was used to predict the nucleotide substitution model showing the best AIC scores. A Maximum Likelihood tree was created using the Rapid Bootstrapping Algorithm, and a search was conducted for the best scoring tree using the General Time Reversible model (GTR+G+I) of molecular evolution and 10,000 bootstrap replicates. A consensus tree was generated with 10% of trees generated early in the chain discarded as burn‐in and a support threshold of 50% for bootstrapping. The threshold for accepting a node as supported is 70%.

## 3 Results

### 3.1 Morphological characterisation

Synonyms: *Vespa ducalis* var. *soror* du Buysson, 1905; *Vespa mandarinia soror* van der Vecht, 1957. Archer (1991) was the first author to consider *Vespa soror* as an independent species.

Material Examined: Two female (workers) specimens from El Campo, Granda (Siero, Asturias, northern Spain), coll. A. Arias, 29 March 2022. One female (worker) specimen from El Campo, Granda (Siero, Asturias, northern Spain), coll. A. Arias, 3 October 2023. One female (worker) specimen from El Campo, Granda (Siero, Asturias, northern Spain), coll. A. Arias, 10 October 2023.

**FIGURE 2:**
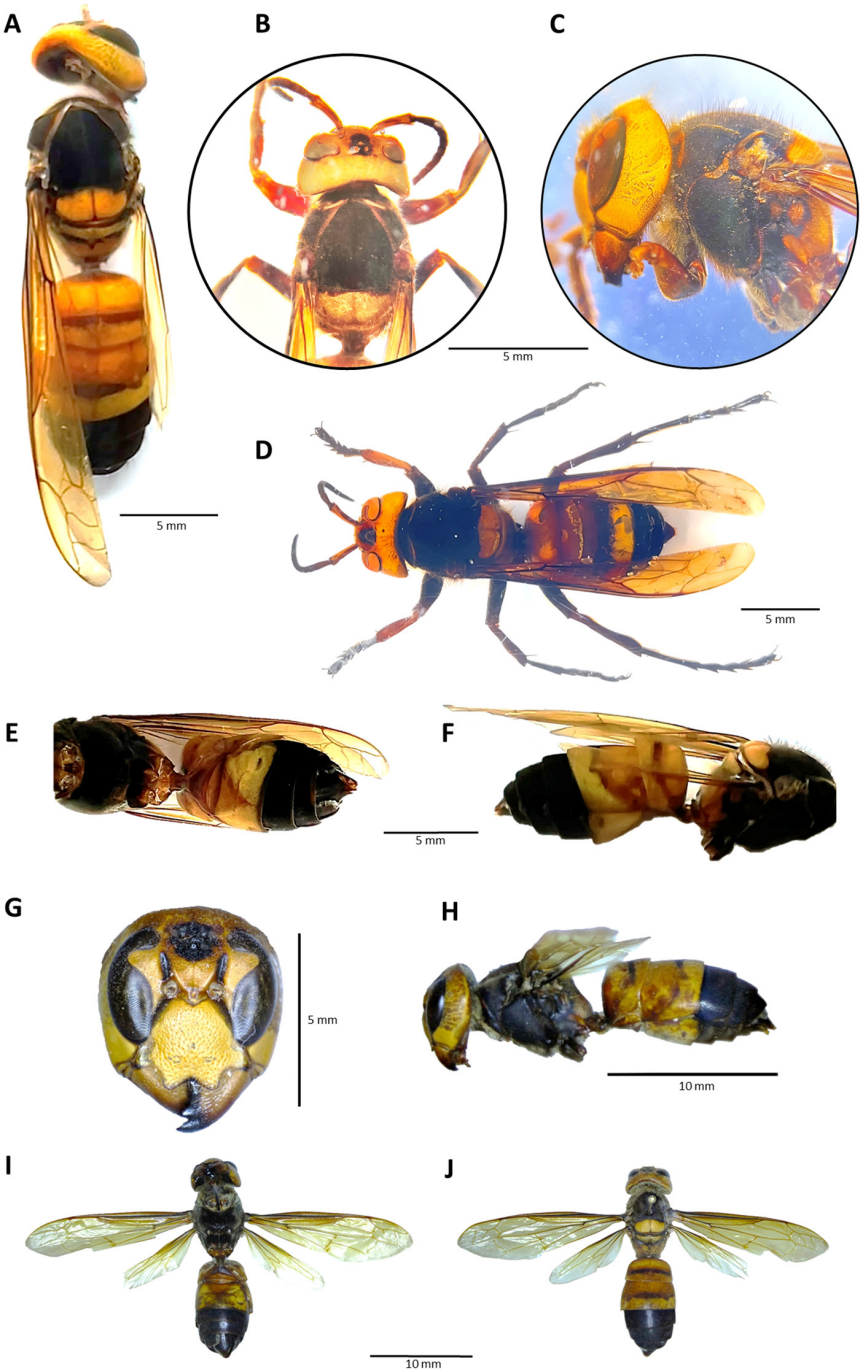
*Vespa soror* specimens trapped in Asturias, northern Spain. Ethanol‐preserved specimens: overall dorsal view (A), detail of the head and thorax, dorsal view (B), detail of the head and thorax, lateral view (C), overall lateral view (D), ventral view (E), lateral view (F), detail of the head of pinned specimen (G), lateral view of the same (H), ventral view of the same (I) and dorsal view of the same (J) of specimens trapped in Asturias, northern Spain.

**FIGURE 3:**
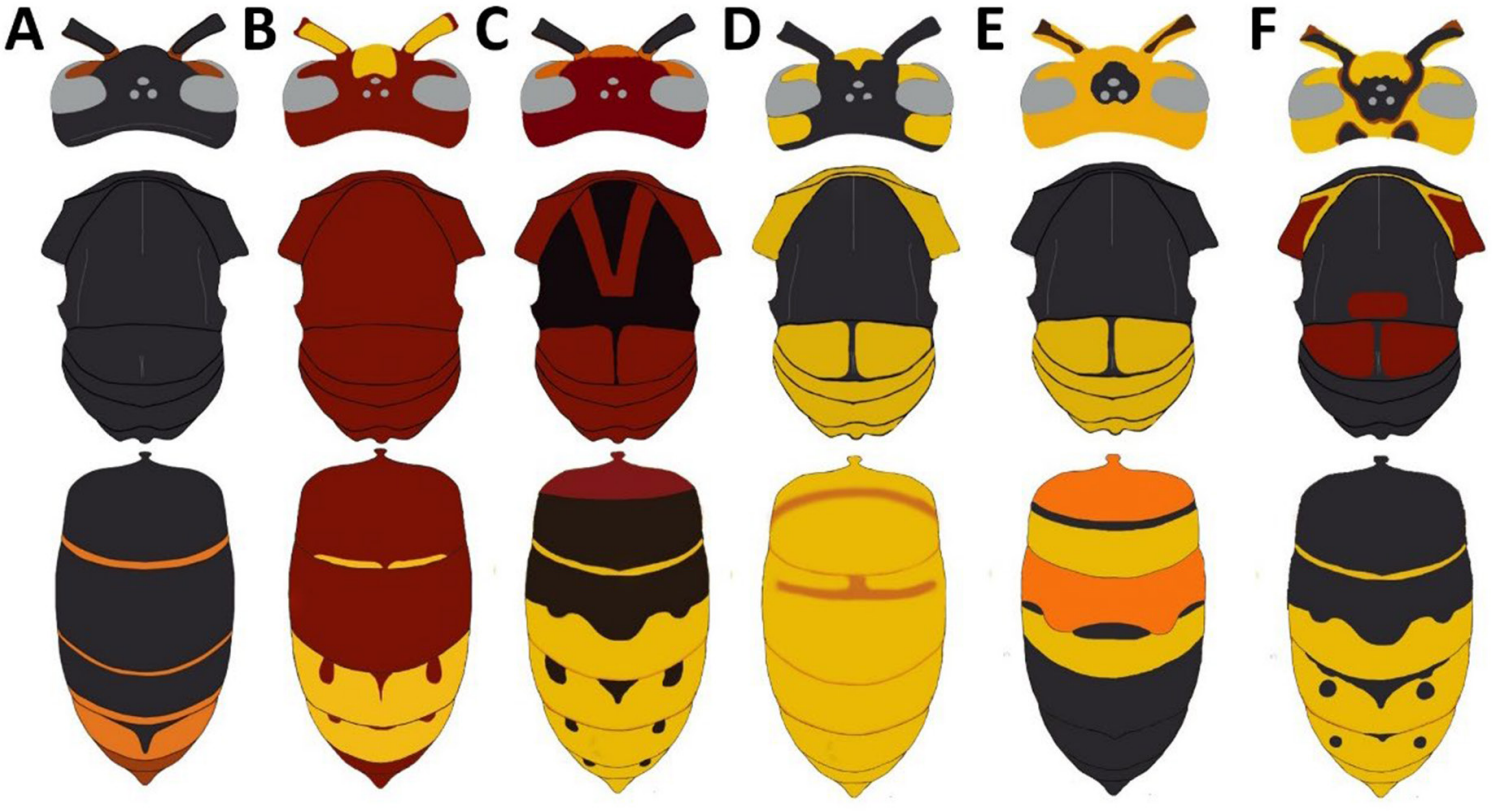
Most common color patterns of the *Vespa* species and *Dolichovespula* of the Iberian Peninsula. *Vespa velutina* (A), *Vespa orientalis* (B), *Vespa crabro* (C), *Vespa bicolor* (D), *Vespa soror* (E), and queen of *Dolichovespula media* (F). Drawings adapted from Perrard et al. (2014).

Description: *Vespa soror* has a peculiar color pattern that is shared by very few Asian species of the genus and is totally unique in the European context. Both sexes are tricolor, with black to blackish‐brown, light‐brown, and yellow areas (Figure 2). The first impression is of an insect with four color blocks, alternatively light and dark. The head, which is exceptionally large, is mostly yellow, followed by a large black to blackish‐brown section comprising most of the mesosoma. A very long section (from the scutellum to the end of T2) that is predominantly yellow and light‐brown but usually bears dark bands or spots, and the terminal segments of the metasoma (practically the posterior half) that are black. Both the antennae and the legs are mostly brown, and the wings are yellowish. *Vespa soror* can be easily distinguished from the other *Vespa* species and gynes of *Dolichovespula media* (Retzius,1783) occurring in Iberia and Europe by looking at the color pattern of the metasoma, since although there are small variations in coloration, they generally present relatively general patterns (Figure 3). Particularly, in *V. soror* the posterior segments of the metasoma are entirely black, while in the rest of native and introduced hornets there are large yellow or orange areas in the posterior half (Figure 3).

Remarks: As indicated by Archer (2012), *V. mandarinia* and *V. soror* females can usually be easily separated from those of other *Vespa* species by the exceptional width of their genae (in lateral view, at the level of the posterior margin of the clypeus, the genal width [GW] is > 1.7 times the eye width [EW], while in the other species it is < 1.3 times; for example, in *Vespa ducalis* the ratio is GW = ca. 1.0 times EW: L. Castro, unpublished data). For their part, the males of *V. mandarinia* and *V. soror* also have rather wide genae (in lateral view, at the level mentioned above, GW is > 1.4 times EW, usually less in the other species) and present a unique combination of other morphological characters, such as the distinctive shape of the aedeagus (apex spade‐shaped, with the basal margin more or less at right angles to the shaft), the structure of the two apical sterna, the eyes, and the antennae (Archer 2012). Then, *V. mandarinia* differs from *V. soror* by a morphological character (the presence of a median impression in the pronotum and the first gastral tergum long as seen from above, about half as long or longer than wide in *V. soror* and less than half as long as wide in *V. mandarinia*) and a color character (T3 to T6 usually entirely black in *V. soror*, T3 to T5 with a narrow or broad orange band, and T6 orange in *V. mandarinia*) (Nguyen et al. 2006). Among the other species of the genus, *Vespa ducalis* Smith, 1852, is practically the only one that can be mistaken for *V. soror* because of an almost identical color pattern: the two species can be separated, among other characters (Nguyen et al. 2006) and, as suggested above, by the width of the genae, which in side view are conspicuously wider than the eyes in *V. soror* (Figure 1a,f) and about the same width as them in *V. ducalis*.

It is often important to be able to determine the caste of the females of social vespids. Body length is not a very reliable character since it depends on variables such as the specimen's position and, if it is dead, on how much it may have shrunk, and then wingspan and wing length are often difficult to assess. In the case of Vespa, various authors have resorted to alternative criteria, such as weight, cuticular chemistry, or ovary development (Pérez de Heredia et al. 2017; Castro and del Pico 2021). However, the latter three criteria pose serious problems of a practical nature, and so some authors have argued for the use of various other morphological clues. Pérez de Heredia et al. (2017) suggested the use of the mesoscutal width as measured between the tegulae: the character is useful because the mesosoma is a part of the body that has a fixed shape, does not shrink when the specimen dies, and can be measured both easily and precisely; also, there appears to be no overlap, so far, between the figures obtained for gynes and workers in the Vespa species examined. More recently, Mattila et al. (2023) have applied to Vespa soror, with this purpose, an array of six characters: head width, forewing length, mesomal width, and the width of the first three terga; of these, based on the measurements they provide for all their reference specimens, we consider only the width of the second tergum (T2) as totally reliable for the separation of gynes and workers, since there is at least some degree of overlap in all the remaining five characters and a good deal of it in three of them, while it is almost non‐existent in T2 width, a character which, besides, has the same practical advantages as intertegular width. The four specimens collected in Asturias are interpreted to be workers, based on the intertegular measurements taken of reference material from five Asian countries (L. Castro, unpublished data) and the maximum width of T2 measured as indicated by Mattila et al. (2023).

### 3.2 Genetic Results

The blast identification engine identified the sequences of the unique sequenced specimen (GenBank: OP.735561 BOLD‐ID: INVA001‐22) as *V. soror* with 98.1% to 97.7% of pairwise identity with four specimens. The remaining hits were with the species *V. crabro* and *V. bicolor,* with a similarity of only 87.8% or less. The BOLD identification procedure revealed 100% and 99.44% of similarity with two sequences identified as *V. soror*. The sequences are marked by BOLD as private, which means that the authors have kept them inaccessible because they are part of as yet unpublished research (Table 1).

**TABLE 1:** BLAST and BOLD identification system (IDS) results for the assembly sequence of the collected specimen.

Species	Similarity (%)	Gen Bank accession	BOLD—ID
*Vespa soror*	100		Private—BOLD
*Vespa soror*	99.4		Private—BOLD
*Vespa soror*	98.1	KF933086	GBAH9518‐14
*Vespa soror*	98		Private—BOLD
*Vespa soror*	97.8	MZ191820	GBMND65039‐21
*Vespa soror*	97.8	MZ191821	GBMND65040‐21
*Vespa soror*	97.7	MZ191821	GBMND65040‐21
*Vespa crabro*	87.8	LC619074	
*Vespa crabro*	87.69		GMGMI008‐14
*Vespa crabro*	87.69		GMGMJ057‐14

**FIGURE 4:**
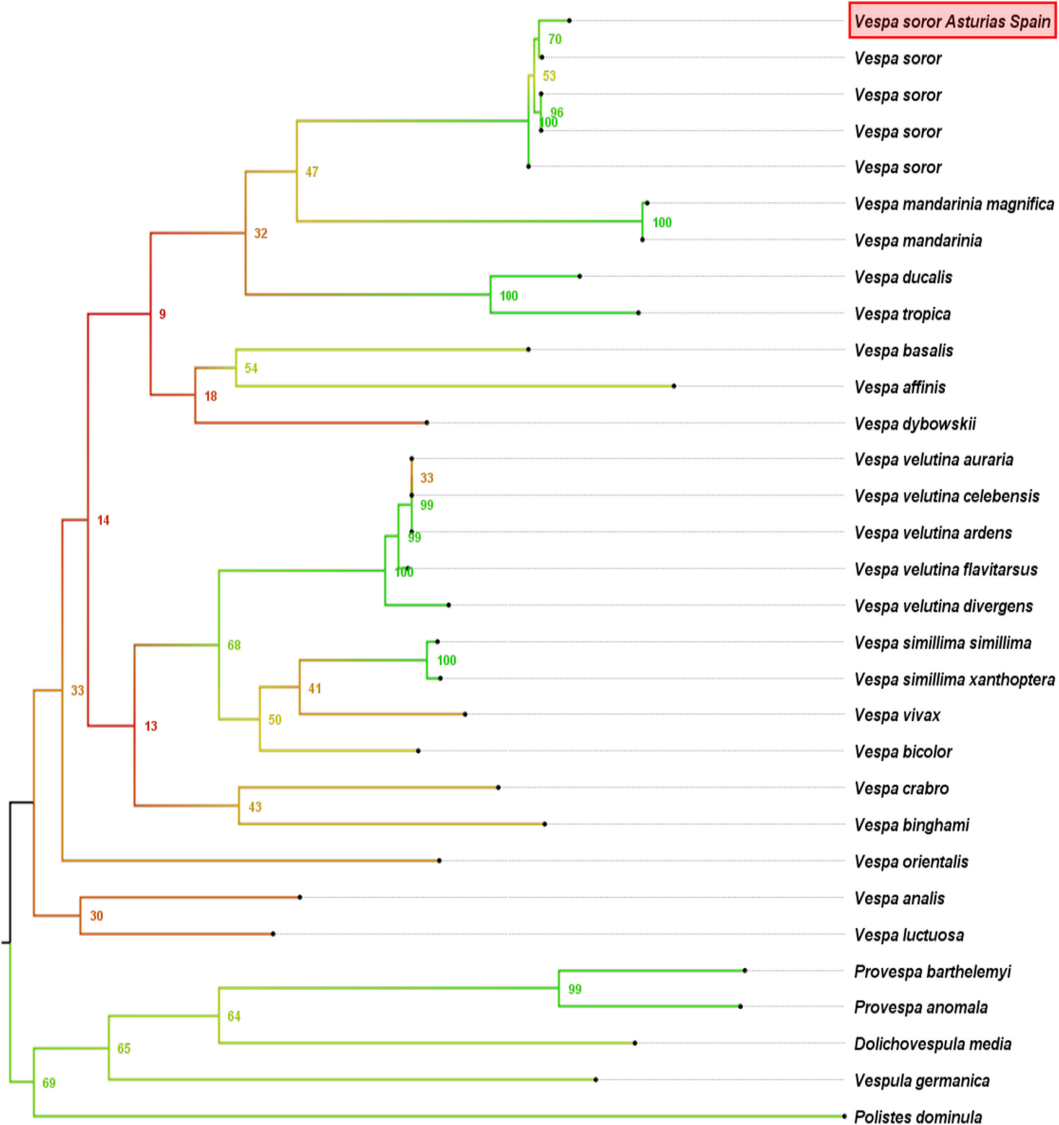
Best Scoring tree from molecular phylogenetic analysis by the Maximum Likelihood method based on COI sequences using the GTR+G+I model with 10.000 bootstraps. The analysis involved 31 nucleotide sequences with a total of 658 positions. GenBank accession numbers are indicated in the Supporting Information, SX.

The phylogenetic analyses clearly show how the sequence of our specimen clusters with the sequences identified in GenBank as *V. soror*. The node clustering them all together has 99.97% bootstrapping support (Figure 4). The distance matrix of the sequences used for the phylogenetic analysis can be found in Supporting Information SY.

## 4 Discussion

*Vespa soror* is an aggressive predator which preys on invertebrates of various sizes, including butterflies, dragonflies, mantids, and grasshoppers, as well as other wasps and even preys on small vertebrates like geckos (Lee 2009). It has even been observed attacking nests of various polistine and vespine wasps such as *Vespa* (*V. bicolor*), *Polistes* and *Parapolybia* (*Pa. indica* (de Saussure, 1854), *Pa. nodosa* van der Vecht, 1966, and *Pa. varia* (Fabricius, 1787)) to capture the larvae and pupae as food for its own young (Lee 2009). Furthermore, *V. soror* has been reported attacking in groups the hives of the honeybee *Apis cerana* Fabricius, 1793, resulting in destroyed hives and causing considerable losses to the beekeeping industry (Lee 2009; Mattila et al. 2020; Cappa et al. 2021). A similar risk may exist in Asturias for hives of *Apis mellifera* Linnaeus, 1758, with potentially greater impacts when considering that the invasive presence of *V. velutina* is already causing serious losses in local honeybee colonies (Requier et al. 2019; Laurino et al. 2020; Cappa et al. 2021).

In the same way, and like the rest of vespid species, *V. soror* can generate problems in the health sector since the sting of *V. soror* is very painful and produces long‐lasting effects. It most likely has a potent venom like *V. tropica* (Linnaeus, 1758) and *V. mandarinia*, both of which are in the same phylogenetic clade, and both are considered to be among the most dangerous stinging insects (Schmidt et al. 1986; Lee 2009). Thus, *V. soror* can become an important cause of medical complications, especially during the months of September to December, the period before the appearance of the males and the new queens when the colonies become large and very defensive (Lee 2009).

Within the endemic distribution of *V. soror*, most colonies are found in forested areas, at altitudes ranging from sea level to over 700 m. The nest is usually located underground, often in the well‐drained soil of a slope, with the entrance hole dug downwards into the soil surface at an angle, and often located among roots of large trees (Lee 2009; Mattila et al. 2023). Some nests are quite close to the surface, just a few centimeters below the ground, but others are deep underground, with one or more tunnels that can be 60 cm or more in length between the nest and the entrance (Lee 2009). In many ways, the nesting behavior of *V. soror* is like that of the closely related *V. mandarinia* (Archer 1995; Lee 2009; Smith‐Pardo, Carpenter, and Kimsey 2020; Bass, Needham, and Bennett 2022; Mattila et al. 2023). This may turn into a problem if this species becomes widespread in northern Spain, since, as in the case of *V. velutina*, the first factor for its effective control is the removal of its nests after detection. Consequently, if *V. soror* nests are underground, this can be an added complication at the time of its search, detection, and elimination by the competent agencies/personnel involved.

In Hong Kong, the queens usually emerge from late March to April each year. Nest initiation probably begins in early May, while the first workers usually appear in June (Lee 2009). Some tentative details on its colony cycle in Vietnam are given by Mattila et al. (2023). These two papers provide us with essential data to elucidate the evolution of the supposed colony or colonies of this species in Asturias. The first two workers detected at the end of March 2022 correspond to an apparently earlier record than observed in their native area. Two things may have happened. That the workers correspond to a very recently formed nest in 2022, which would imply that the hibernation emergence has been advanced. It is also possible that they correspond to workers from a nest created in 2021 and that they have endured the winter. The climatic conditions of their native area are not different from those observed in Asturias during the spring months of 2022; this climate, temperate and increasingly less cold due to climate change, may have favored and in turn altered the nesting period of *V. soror*, as has already been observed in our region with *V. velutina*. On the other hand, having found two workers in early October 2023 indicates that at least one gyne reared in 2022 has been able to survive the winter to form a new colony in 2023.

As a possible introduction pathway of this species, we believe that it is most likely to have arrived during hibernation as a stowaway from one of the countries where it is native, as has already happened with other hornet species, such as *V. mandarinia* or *V. velutina* (Otis, Taylor, and Mattila 2023).

Recently the Entomological Society of America proposed, as a common name for *V. soror*, “southern giant hornet,” and the term has been quickly accepted and has spread worldwide, both in English and in translations to other languages. On the Internet, there is already at least one Spanish version, “*avispón gigante del sur*,” and the other possible version, “*avispón gigante meridional*,” may be in use as well. Those terms are descriptive and precise, and consequently perfect for the academic community, but they may not be ideal for the press, the Internet, and popular science because they are relatively long and therefore cumbersome for such “everyday” uses, and it is easy to imagine that people may easily end up shortening them to “*avispón gigante*” (giant hornet) and thus leading to confusion with *V. mandarinia* (the northern giant hornet), or avoiding them altogether and turning to such undesirable terms as the Spanish versions of “Asian hornet” or even “murder hornet”.

So, it seems essential to have an additional Spanish term that might be as precise as its longer equivalents but at the same time “non‐shortenable” and convenient for its everyday use on the Internet, the press, and so on. A new name is accordingly proposed here, “*avispón sóror,*” which checks all those boxes: it is short, convenient, and unmistakable, and then it provides an unequivocal association with the scientific name, in the tradition of other Spanish terms in current use, “*avispa velutina*,” “*avispón bicolor*,” or “*avispón oriental*/*is*”.

## 5 Conclusions

The first records of *V. soror* in Europe are reported and supported by morphological and genetic analyses. This record brings to four the number of *Vespa* species introduced to the Iberian Peninsula.

The arrival of this species is probably linked to the transport as a stowaway from one of the countries where it is native. Its presence in the northern region of Iberia, where the invasive *V. velutina* is widespread, could have a cumulative effect increasing the environmental, economic, and even public health damage already triggered by *V. velutina*.

Its presence, although still scarce, is at a critical point for its eradication and correct management through the implementation of a rapid response plan in the location of its nests. In the next few years, monitoring activities will be strongly increased (also involving neighboring areas of Siero) and will be aimed at verifying the presence and potential spread of the species in the region, as well as at assessing possible damage and implications of the arrival of another invasive hornet species in northern Spain in the beekeeping, ecological, and even sanitary sectors.

## Author Contributions

**Omar Sánchez:** data curation (equal), formal analysis (lead), investigation (equal), methodology (equal), writing – original draft (lead), writing – review and editing (equal). **Leopoldo Castro:** formal analysis (equal), investigation (equal), writing – original draft (equal), writing – review and editing (equal). **Álvaro Fueyo:** formal analysis (equal), investigation (equal), writing – review and editing (equal). **Yaisel J. Borrell:** resources (equal), writing – review and editing (equal). **Andrés Arias:** conceptualization (lead), formal analysis (equal), funding acquisition (lead), investigation (equal), methodology (equal), supervision (lead), writing – original draft (equal), writing – review and editing (lead).

## Conflicts of Interest

The authors declare no conflicts of interest.

## Supporting information


**Appendix S1.**.

**Appendix S2.**.

## Data Availability

All the genetic data is available at GenBank using the accession numbers provided in text, Table 1 and the Supporting Information.

## References

[ece370502-bib-0001] Arca, M. , F. Mougel , T. Guillemaud , et al. 2015. “Reconstructing the Invasion and the Demographic History of the Yellow‐Legged Hornet, *Vespa velutina*, in Europe.” Biological Invasions 17: 2357–2371. 10.1007/s10530-015-0880-9.

[ece370502-bib-0002] Archer, M. E. 1991. “The Number of Species That Can Be Recognised Within the Genus *Vespa* (Hym., Vespinae).” Entomologist's Monthly Magazine 127: 161–164.

[ece370502-bib-0003] Archer, M. E. 1995. “Taxonomy and Bionomics of the *Vespa mandarinia* Group (Hym., Vespinae).” Entomologist's Monthly Magazine 131: 47–53.

[ece370502-bib-0004] Archer, M. E. 2012. Vespine Wasps of the World. Behaviour, Ecology and Taxonomy of the Vespinae, 1–352. Manchester: Siri Scientific Press.

[ece370502-bib-0006] Bass, A. , K. Needham , and A. M. Bennett . 2022. “First Record of *Vespa crabro* Linnaeus (Hymenoptera: Vespidae) in Western North America With a Review of Recorded Species of *Vespa* Linnaeus in Canada.” Zootaxa 5154, no. 3: 305–318. 36095620 10.11646/zootaxa.5154.3.4

[ece370502-bib-0007] Cappa, F. , A. Cini , L. Bortolotti , J. Poidatz , and R. Cervo . 2021. “Hornets and Honey Bees: A Coevolutionary Arms Race Between Ancient Adaptations and New Invasive Threats.” Insects 12: 1037. 10.3390/insects12111037. 34821837 PMC8625458

[ece370502-bib-0008] Castro, L. 2019. “Una Nueva Introducción Accidental en el Género *Vespa*: *Vespa Bicolor* en la Provincia de Málaga (España).” Revista Gaditana de Entomología 10: 47–56.

[ece370502-bib-0009] Castro, L. , and C. del Pico . 2021. “Sobre el problema de *Vespa orientalis* (Hymenoptera: Vespidae) en el sur de España.” Revista Gaditana de Entomología 12: 183–206.

[ece370502-bib-0010] Castro, L. , and S. Pagola‐Carte . 2010. “ *Vespa velutina* (Hymenoptera: Vespidae), recolectada en la Península Ibérica.” Heteropterus Revista de Entomología 10: 193–196.

[ece370502-bib-0011] Daglio, A. 2019. On the Taxonomy and Distribution of the Subfamily Vespinae, 1–49. Mauritius: Lambert Academic Publishing.

[ece370502-bib-0012] Fajardo, M. C. , and I. Sánchez . 2020. “Ciencia ciudadana, globalización y especies invasoras. El caso del avispón oriental, *Vespa orientalis* en Algeciras.” Almoraima, Revista de Estudios Campogibraltareños 52: 233–237.

[ece370502-bib-0013] Folmer, O. , M. Black , W. Hoeh , R. Lutz , and R. Vrijenhoek . 1994. “DNA Primers for Amplification of Mitochondrial Cytochrome C Oxidase Subunit I From Diverse Metazoan Invertebrates.” Molecular Marine Biology and Biotechnology 3: 294–299. 7881515

[ece370502-bib-0014] Girish‐Kumar, P. , and G. Srinivasan . 2010. “ taxonomic Studies of Hornet Wasps (Hymenoptera: Vespidae) *Vespa* of India.” Records of the Zoological Survey of India 110: 57–80. 10.26515/rzsi/v110/i2/2010/158949.

[ece370502-bib-0015] Grosso‐Silva, J. M. , and M. Maia . 2012. “ *Vespa velutina* (Hymenoptera, Vespidae), New Species for Portugal.” Arquivos Entomolóxicos 6: 53–54.

[ece370502-bib-0016] Hernández, R. , F. J. García‐Gans , J. Selfa , and J. Rueda . 2013. “Primera Cita de la Avispa Oriental Invasora *Vespa orientalis* (Hymenoptera: Vespidae) en la Península Ibérica.” Boletín de la Sociedad Entomológica Aragonesa 52: 299–300.

[ece370502-bib-0017] Kozak, P. , and G. W. Otis . 2020. “From the Province. New Honey Bee Pests in North America: Asian Hornets Reported and Confirmed in British Columbia.” Ontario Bee Journal 39: 20–21.

[ece370502-bib-0018] Kurzenko, N. V. 1995. “Sem. Vespidae. skladchatokrylye osy.” In Opreditel’ nasekomykh Dal'nego Vostoka Rossii, edited by P. D. Ler , 1–608. St. Peterburg: Nauka.

[ece370502-bib-0019] Laurino, D. , S. Lioy , L. Carisio , A. Manino , and M. Porporato . 2020. “ *Vespa velutina*: An Alien Driver of Honey Bee Colony Losses.” Diversity 12: 5. 10.3390/d12010005.

[ece370502-bib-0020] Lee, J. X. 2009. “A Note on *Vespa soror* (Hymenoptera: Vespidae) in Hong Kong.” Hong Kong Entomological Bulletin 1: 18–22.

[ece370502-bib-0021] Lee, J. X. 2010. “Notes on *Vespa analis* and *Vespa mandarinia* (Hymenoptera, Vespidae) in Hong Kong, and a Key to all *Vespa* Species Known From the SAR.” Hong Kong Entomological Bulletin 2: 31–36.

[ece370502-bib-0022] Lioy, S. , C. Bergamino , and M. Porporato . 2022. “The Invasive Hornet *Vespa velutina*: Distribution, Impacts and Management Options.” CABI Reviews: 1643–1649. 10.1079/cabireviews202217030.

[ece370502-bib-0023] López, S. , M. González , and A. Goldarazena . 2011. “ *Vespa velutina* Lepeletier, 1836 (Hymenoptera: Vespidae): First Records in Iberian Peninsula.” EPPO Bulletin 41: 439–441. 10.1111/j.1365-2338.2011.02513.x.

[ece370502-bib-0024] Mattila, H. R. , L. T. P. Nguyen , A. Perrard , M. Bain , and G. W. Otis . 2023. “Biology of the Southern Giant Hornet, Vespa Soror: Nest Architecture, Morphological Differences Among Castes, and the Genetic Structure of Colonies.” Frontiers in Insect Science 3: 1136297. 10.3389/finsc.2023.1136297. 38469522 PMC10926378

[ece370502-bib-0025] Mattila, H. R. , G. W. Otis , L. T. P. Nguyen , H. D. Pham , O. M. Knight , and N. T. Phan . 2020. “Honey Bees (*Apis cerana*) use Animal Feces as a Tool to Defend Colonies Against Group Attack by Giant Hornets (*Vespa soror*).” PLoS ONE 15: e0242668. 10.1371/journal.pone.0242668. 33296376 PMC7725375

[ece370502-bib-0026] Monceau, K. , O. Bonnard , and D. Thiéry . 2014. “ *Vespa velutina*: A New Invasive Predator of Honeybees in Europe.” Journal of Pest Science 87: 1–16. 10.1007/s10340-013-0537-3.

[ece370502-bib-0027] Nguyen, L. T. , F. Saito , J. I. Kojima , and J. M. Carpenter . 2006. “Vespidae of Viet Nam (Insecta: Hymenoptera) 2.” Taxonomic Notes on Vespinae. Zoological Science 23, no. 1: 95–104. 16547411 10.2108/zsj.23.95

[ece370502-bib-0028] Noll, F. B. , M. da Silva , L. A. Oliveira , and S. Mateus . 2020. “Caste: Social Wasps.” In Encyclopedia of Social Insects, edited by C. K. Starr , 1–246. Cham: Springer. 10.1007/978-3-319-90306-4_140-1.

[ece370502-bib-0029] Otis, G. W. , B. A. Taylor , and H. R. Mattila . 2023. “Invasion Potential of Hornets (Hymenoptera: Vespidae: *Vespa* Spp.).” Frontiers in Insect Science 3: 1145158. 38469472 10.3389/finsc.2023.1145158 PMC10926419

[ece370502-bib-0030] Pérez de Heredia, I. , E. Darrouzet , A. Goldarazena , P. Romón , and J. C. Iturrondobeitia . 2017. “Differentiating Between Gynes and Workers in the Invasive Hornet *Vespa velutina* (Hymenoptera, Vespidae) in Europe.” Journal of Hymenoptera Research 60: 119–133. 10.3897/jhr.60.13505.

[ece370502-bib-0031] Perrard, A. , M. Arca , Q. Rome , et al. 2014. “Geographic Variation of Melanisation Patterns in a Hornet Species: Genetic Differences, Climatic Pressures or Aposematic Constraints?” PLoS ONE 9: e94162. 10.1371/journal.pone.0094162. 24740142 PMC3989226

[ece370502-bib-0032] Requier, F. , Q. Rome , G. Chiron , et al. 2019. “Predation of the Invasive Asian Hornet Affects Foraging Activity and Survival Probability of Honey Bees in Western Europe.” Journal of Pest Science 92: 567–578. 10.1007/s10340-018-1063-0.

[ece370502-bib-0033] Sánchez, I. , M. C. Fajardo , and M. Castro . 2019. “Primeras citas del avispón oriental *Vespa orientalis* (Hymenoptera: Vespidae) para Andalucía (España).” Revista de la Sociedad Gaditana de Historia Natural 13: 11–14.

[ece370502-bib-0034] Sanger, F. , and A. R. Coulson . 1975. “A Rapid Method for Determining Sequences in DNA by Primed Synthesis With DNA Polymerase.” Journal of Molecular Biology 94: 441–448. 10.1016/0022-2836(75)90213-2. 1100841

[ece370502-bib-0035] Schmidt, J. O. , S. Yamane , M. Matsuura , and C. K. Starr . 1986. “Hornet Venoms: Lethalities and Lethal Capacities.” Toxicon 24: 950–954. 10.1016/0041-0101(86)90096-6. 3810666

[ece370502-bib-0036] Smith‐Pardo, A. H. , J. M. Carpenter , and L. Kimsey . 2020. “The Diversity of Hornets in the Genus *Vespa* (Hymenoptera: Vespidae, Vespinae), their Importance and Interceptions in the United States.” Insect Systematics and Diversity 4: 1–27. 10.1093/isd/ixaa006.

[ece370502-bib-0039] Taylor B. A. , L. R. Tembrock , M. Sankovitz , et al. 2024. “Population genomics of the invasive Northern Giant Hornet Vespa mandarinia in North America and across its native range.” Scientific Reports 14, no. 3: 10803. 38734771 10.1038/s41598-024-61534-0 PMC11088652

[ece370502-bib-0037] Vega, J. M. , F. J. Ortiz‐Sánchez , A. Martínez‐Arcediano , et al. 2022. “Social Wasps in Spain: The Who and Where.” Allergologia et Immunopathologia 50: 58–64. 10.15586/aei.v50i2.523. 35257546

[ece370502-bib-0038] Villemant, C. , M. Barbet‐Massin , A. Perrard , et al. 2011. “Predicting the Invasion Risk by the Alien Bee‐Hawking Yellow‐Legged Hornet *Vespa velutina nigrithorax* Across Europe and Other Continents With Niche Models.” Biological Conservation 144: 2142–2150. 10.1016/j.biocon.2011.04.009.

